# A Child With Barking Cough

**DOI:** 10.1016/j.acepjo.2025.100085

**Published:** 2025-02-21

**Authors:** Abdullah Khan, Hassan Baghazal

**Affiliations:** 1 Department of Emergency Medicine, Sidra Medicine, Doha, Qatar; 2 Pediatric Surgery, Sidra Medicine, Doha, Qatar

**Keywords:** barking cough, foreign body aspiration, pediatrics

## 1 Case Presentation

**Figure 1:**
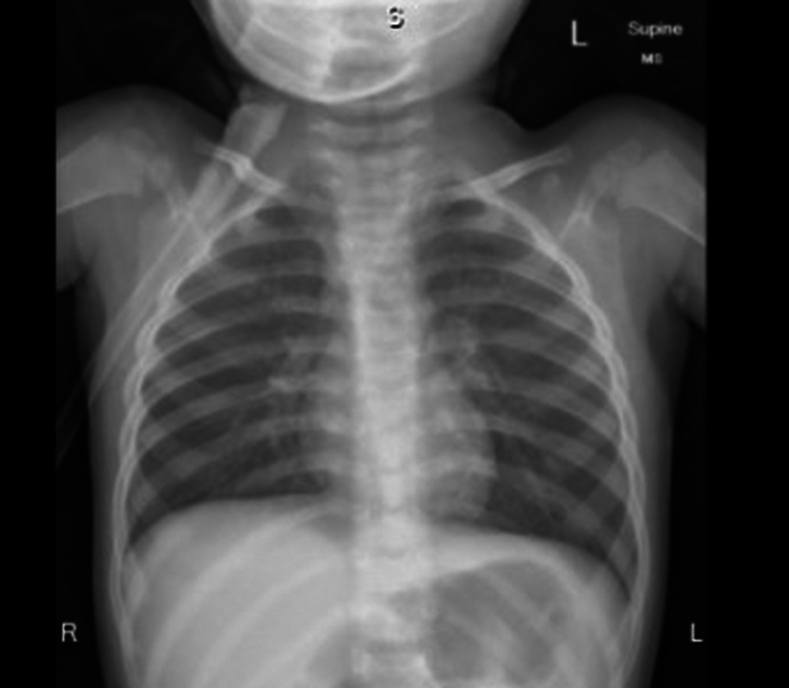
X-ray chest shows mild hyperinflation of left lung.

A 13-month-old child was referred to our emergency department from an outside hospital with a case of barking cough for 3 days associated with difficulty in breathing and fever not improving with antibiotics, albuterol and epinephrine nebulization. As per history, the child initially presented to the outside hospital 3 days ago as a case of sudden onset of coughing spell while playing in the kitchen, followed by an episode of cyanosis lasting for a few seconds that self-resolved. There was no witnessed choking episode of foreign bodies. The patient was treated as a case of acute life-threatening event, observed for 1 day in the hospital, and discharged. Two days later the patient presented again to the outside hospital with fever, barking cough, inspiratory stridor, and difficulty in breathing. The patient was treated as a case of croup with dexamethasone and nebulized epinephrine with little improvement. At this point, the patient was transferred to our emergency department for further evaluation. On initial evaluation, the patient had a barking cough, hoarse voice, and inspiratory stridor. On auscultation, there was decreased air entry bilaterally associated with wheezes. An additional dose of dexamethasone, nebulized epinephrine, and albuterol were administered with partial improvement. A chest x-ray was obtained, which showed mild hyperinflation of the left side raising suspicion of radiolucent foreign body aspiration (Fig 1).

**Figure 2:**
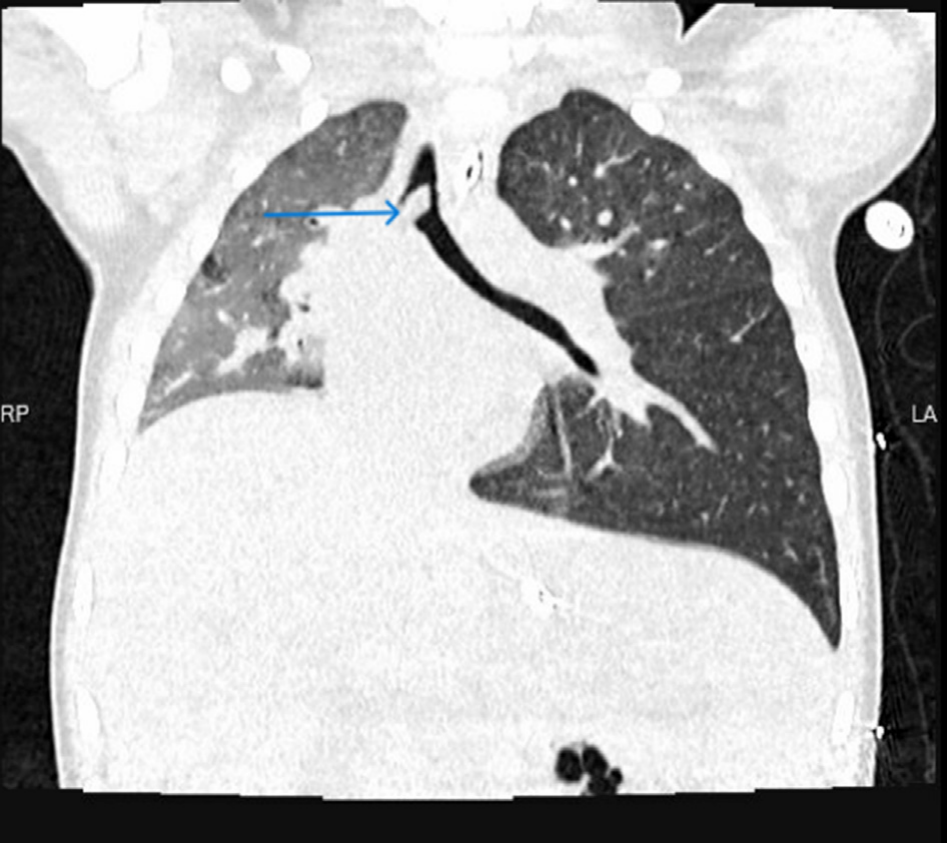
Chest CT scan (coronal view) shows foreign body at level of carina extending to right bronchus (blue arrow). CT, computed tomography.

**Figure 3:**
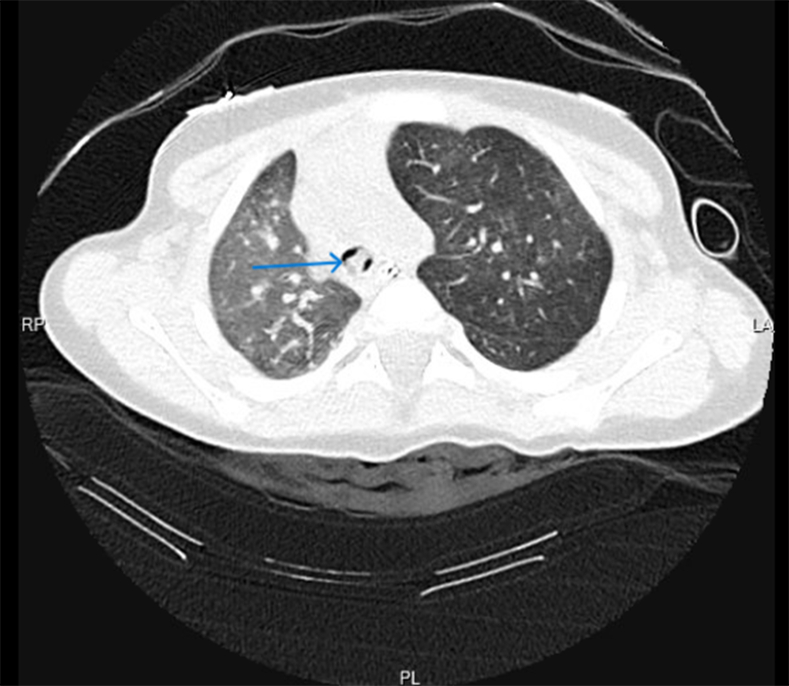
Chest CT scan (cross-sectional view) shows foreign body (blue arrow). CT, computed tomography.

**Figure 4:**
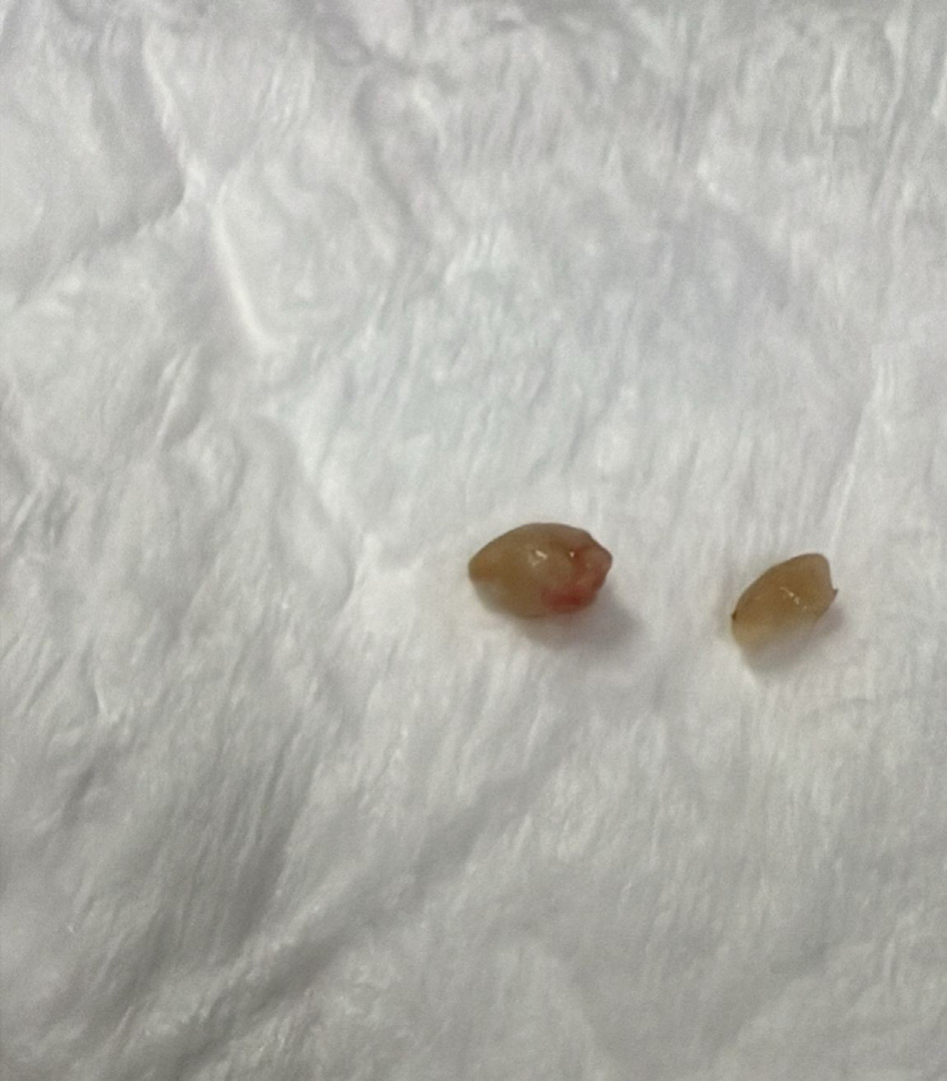
Two pieces of peanuts retrieved by bronchoscopy.

During stay in the emergency department, the patient developed hypoxia (oxygen saturations of 90%-91% on room air) and signs of respiratory distress (retractions of intercostal and suprasternal muscles). A venous blood gas showed respiratory acidosis (pH: 7.14, PCO_2_: 85 mm Hg). The patient was intubated and mechanically ventilated. A computed tomography (CT) scan of the chest was obtained to evaluate any aspirated foreign body. The CT chest showed a tracheal foreign body at the level of the carina extending to the right main stem bronchus (Figs 2 and 3; blue arrows). The foreign body had caused partial collapse of the right lung and hyperinflation of the left lung. Pediatric surgery was consulted. The patient was taken to the operating room for rigid bronchoscopy, and 2 pieces of peanuts were retrieved (Fig 4). The patient recovered completely without any complications and was discharged home.

## 2 Diagnosis: Foreign Body Aspiration

Foreign body aspiration is common in toddlers. The most common aspirated foreign bodies are organic in nature, predominantly peanuts. Common symptoms and signs include witnessed choking, coughing, wheezing, and decreased air entry on auscultation. The most common finding on chest radiograph is air trapping.^1^ Normal physical examination and normal chest radiograph do not exclude the presence of foreign body. The diagnostic yield of physical findings and chest radiographs increases 24 hours after the event.^2^ Often there can be delays in diagnosing foreign body aspirations. The most reasons for delay are parental negligence, misdiagnosis as clinical findings can overlap with other respiratory ailments (eg, asthma, pneumonia, etc), and reliance on normal chest radiographs.^3^

Because it is a challenging diagnosis, various clinical models have been developed to identify clinical features that can predict foreign body aspiration in children. In a meta-analysis of different models performed by Lee et al,^4^ focal hyperinflation on chest radiograph (odds ratio [OR]; 8.3), unilateral decreased air entry (OR; 4.8), witnessed choking (OR; 3.1), and wheezing (OR; 2.5) were strongly associated with diagnosis of foreign body aspiration. In conclusion, diagnosis of foreign body aspiration can be challenging. A combination of history, physical examination, and radiographic findings can provide useful clues for diagnosing foreign body aspirations.

## Conflict of Interest

All authors have affirmed they have no conflicts of interest to declare.
